# A dataset on the species composition of amphipods (Crustacea) in a Mexican marine national park: Alacranes Reef, Yucatan

**DOI:** 10.3897/BDJ.6.e22622

**Published:** 2018-01-25

**Authors:** Carlos E. Paz-Ríos, Nuno Simões, Daniel Pech

**Affiliations:** 1 Laboratorio de Biodiversidad Marina y Cambio Climatico (BIOMARCCA), El Colegio de la Frontera Sur, Unidad Campeche, Lerma, Campeche, Mexico; 2 Unidad Academica Sisal, Facultad de Ciencias, Universidad Nacional Autonoma de Mexico, Puerto de Abrigo, Sisal, Yucatan, Mexico; 3 Laboratorio Nacional de Resiliencia Costera (LANRESC), Puerto de Abrigo, Sisal, Yucatan, Mexico; 4 International Chair for Ocean and Coastal Studies, Harte Research Institute, Texas A&M, Corpus Christi, Texas, United States of America

**Keywords:** Amphipoda, Peracarida, Macrofauna, Benthos, Species diversity, Coral Reef, Campeche Bank, Gulf of Mexico, occurrence record

## Abstract

**Background:**

Alacranes Reef was declared as a National Marine Park in 1994. Since then, many efforts have been made to inventory its biodiversity. However, groups such as amphipods have been underestimated or not considered when benthic invertebrates were inventoried. Here we present a dataset that contributes to the knowledge of benthic amphipods (Crustacea, Peracarida) from the inner lagoon habitats from the Alacranes Reef National Park, the largest coral reef ecosystem in the Gulf of Mexico. The dataset contains information on records collected from 2009 to 2011. Data are available through Global Biodiversity Information Facility (GBIF).

**New information:**

A total of 110 amphipod species distributed in 93 nominal species and 17 generic species, belonging to 71 genera, 33 families and three suborders are presented here. This information represents the first online dataset of amphipods from the Alacranes Reef National Park. The biological material is currently deposited in the crustacean collection from the regional unit of the National Autonomous University of Mexico located at Sisal, Yucatan, Mexico (UAS-Sisal). The biological material includes 588 data records with a total abundance of 6,551 organisms. The species inventory represents, until now, the richest fauna of benthic amphipods registered from any discrete coral reef ecosystem in Mexico.

## Introduction

Alacranes Reef is a highly diverse protected coral reef ecosystem, located in the southern sector of the Gulf of México. It is considered as one of the largest coral reefs of the Gulf of Mexico (Liceaga-Correa and Hernández-Núñez 2000, Tunnell et al. 2007a). It was declared as a National Marine Park by the Mexican government in 1994 and as a Biosphere Reserve, by the UNESCO, in 2006. Since its declaration as a National Park, Alacranes Reef has been subject to different efforts to inventory its biodiversity, however, this task is not fulfilled until now. Recent studies on reef-associated benthic fauna include sea anemones (González-Muñoz et al. 2013), decapod crustaceans (Santana-Moreno et al. 2013), polychaetes (Ramírez-Hernández et al. 2015), sponges (Ugalde et al. 2015) and mollusks (Reyes-Gómez et al. 2017).The peracarid crustaceans, such as the amphipods, are one of the underestimated groups not considered in the main inventory lists reported for the reef (CONANP 2006, Tunnell et al. 2007b). The scarce evidence describing the richness of amphipods on the Alacranes reef (Paz-Ríos et al. 2014, Paz-Ríos et al. 2013a, Paz-Ríos et al. 2013b) has pointed out the relevance of this group as one of the most diverse in the entire reef ecosystem. The present contribution intends to show the high species richness of benthic amphipods inhabiting the bottom habitats from the inner reef lagoon by providing a dataset with taxonomic checklist. This checklist constitutes the first formal effort to inventory the biodiversity of such group in the Alacranes Reef ecosystem.

## Project description

### Title

Alacranes Reef Biodiversity Expedition.

### Personnel

Carlos Enrique Paz Ríos and Nuno Simões.

### Design description

The dataset was composed using information from benthic macrofauna samples randomly collected on different benthic habitats and artificial substrates between 2009 and 2011, mostly in the inner lagoon of Alacranes reef.

### Funding

Resources included funds to NS from the National Autonomous University of Mexico (UNAM) for supporting academic research (PAPIME-PE207210), funds from the Mexican Government for scientific research (SEMARNAT-CONACyT108285) and an international cooperation for marine scientific research between Mexico and USA (HRI-CONABIO-NE018) through the project “Actualización del conocimiento de la diversidad de especies de invertebrados marinos bentónicos de aguas someras (<50 m) del Sur del Golfo de México”. Funds to CEPR was provided by CONACyT scholarship.

## Sampling methods

### Study extent

The dataset contains information on amphipods collected from three different expeditions: the first one in August 2009, the second one in April 2010 and the third one in October 2011. In each expedition a relatively high diversity of habitats was explored in order to increase the chance to capture the species diversity. In the 2010 and 2011 expeditions, special effort was made to collect information on navigation buoys and two wood docks nearby a sand island (isla Perez).

### Sampling description

Amphipods were collected from 50 sampling sites: Forty-one randomly distributed on natural habitats from the inner lagoon, including different benthic habitats (i.e. bare substrate, seagrass bed, coral patch, reef wall) and nine collected on artificial substrates (i.e. buoy, wood dock, lobster tramp). Diverse sampling devices were employed: PVC core for sandy substrates, shovel for patches of intertidal sand, suction device sampler for coral rubble, sand and shallow coral reef flats and, finally, a Riley push-net and trawl for shallow seagrass beds of *Thalassia testudinum* (Banks ex König, 1805).

### Quality control

Amphipod species were sorted and identified to the lowest possible taxonomical category, using specialised literature such as keys for identification, e.g. Myers (1981), Lowry and Stoddart (1997), LeCroy (2000) and illustrated guides, e.g. Winfield et al. (2007), Ortiz et al. (2014). The taxonomical arrangement used herein follows Lowry and Myers (2017). Nine species were identified as possibly new species and were represented at the genus category (i.e. *Bemlos*, *Elasmopus*, *Idunella*, *Harpinia*, *Leucothoe*, *Maera*, *Psammogammarus*); further specialised taxonomical effort will be needed before assigning a name. Species names were matched using the *Taxon Match* tool in the World Register of Marine Species (WoRMS) in order to corroborate, standardise and update if necessary. Sampling points in the reef, available in the Data resources (see below), were georeferenced and displayed into different maps using different software (i.e. QGIS^®^, ArcView GIS^®^, and Google Maps^®^).

### Step description

Once collected, the amphipods were first anaesthetised with magnesium chloride (4%) for 15 to 20 min. and then fixed with formaldehyde solution (10%) buffered with seawater before storage. In the laboratory, samples were washed through a 500 µm size mesh and preserved with 70% of ethanol solution. Finally, the organisms were stored in glass containers with a catalogue number according to the regional collection ‘Crustáceos de Yucatán’ (YUC-CC-255-11), from the ‘Universidad Nacional Autónoma de México, Unidad Sisal’ Mexico.

## Geographic coverage

### Description

**Figure 1.**
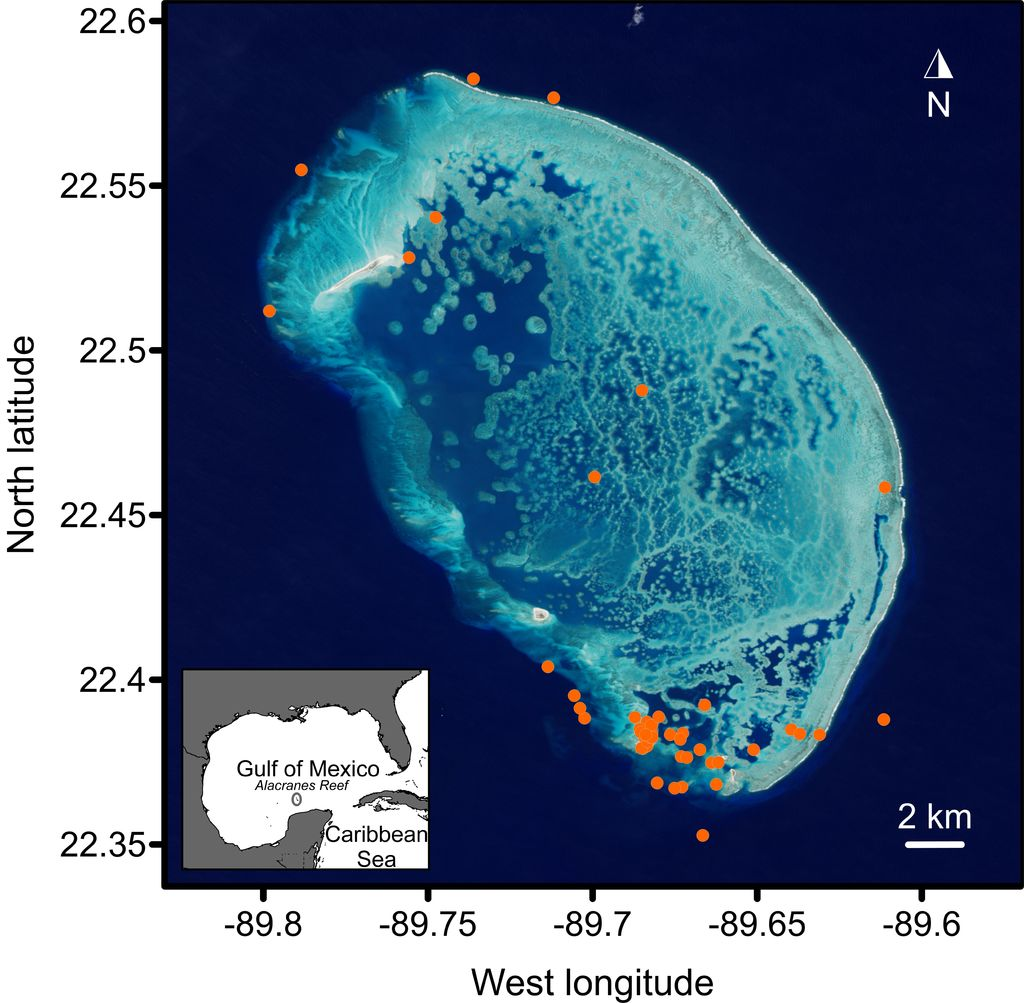
Location of sampling points in the Alacranes Reef National Park. The image from the NASA Earth Observatory, was acquired by Jesse Allen on November 5, 2014, by the Operation Land Imager on Landsat 8.

The Alacranes Reef, Yucatan, Mexico (Fig. 1), is located in the Campeche/Yucatanean Outer Neritic area (Wilkinson et al. 2009) forming part of the North America's marine ecoregion 14 (Southern Gulf of Mexico). It is a platform reef type with a semi-elliptic shape, covering an area larger than 650 km^2^ (Liceaga-Correa and Hernández-Núñez 2000).

### Coordinates

22.337 and 22.605 North Latitude; -89.549 and -89.852 West Longitude.

## Taxonomic coverage

### Description

The dataset contains information on 110 species (93 nominal species and 17 generic species), belonging to 71 genera, 33 families and three suborders. The suborder Amphilochidea was composed of 46 species, 28 genera and 13 families, the suborder Colomastigidea by 6 species, 1 genus and 1 family and the suborder Senticaudata by 58 species, 42 genera and 19 families (Table 1). Regardless suborder, 6 families presented the highest numbers of species, accounting for the 50% of all the fauna in the reef: Ampithoidae (6 spp.), Colomastigidae (6 spp.), Lysianassidae (8 spp.), Leucothoidae (11 spp.), Maeridae (12 spp.) and Aoridae (13 spp.).

## Temporal coverage

**Data range:** 2009-8-01 – 2011-10-17.

## Usage rights

### Use license

Other

### IP rights notes

Creative Commons Attribution Non Commercial (CC-BY-NC) 4.0 License

## Data resources

### Data package title

Benthic amphipods from Alacranes Reef, Campeche Bank, Mexico

### Resource link

http://ipt.iobis.org/caribbeanobis/resource?r=anfipodosbentonicos_bancodecampeche_mexico

### Alternative identifiers

http://ipt.iobis.org/caribbeanobis/archive.do?r=anfipodosbentonicos_bancodecampeche_mexico

### Number of data sets

1

### Data set 1.

#### Data set name

Benthic amphipods from Alacranes Reef, Campeche Bank, Mexico

#### Data format

Darwin Core Archive (DwC-A)

#### Number of columns

15

#### Character set

UTF-8

#### Data format version

1.0

#### Description

The dataset presents an occurrence data sheet with 15 columns including information for 588 records.

**Data set 1.** 

Column label	Column description
eventID	An identifier for the set of information associated with an Event (something that occurs at a place and time).
basisOfRecord	The specific nature of the data record.
occurrenceID	An identifier for the Occurrence (i.e. number of collection catalogue).
individualCount	The number of individuals represented present at the time of the Occurrence.
occurrenceStatus	A statement about the presence or absence of a Taxon at a Location.
associatedReferences	An identifier (i.e. Digital Object Identifier, DOI) of literature associated with the Occurrence.
samplingProtocol	The name of, reference to, or description of the method or protocol used during an Event.
eventDate	The date-time or interval during which an Event occurred.
decimalLatitude	The geographic latitude (in decimal degrees, using the spatial reference system given in geodeticDatum) of the geographic centre of a Location.
decimalLongitude	The geographic longitude (in decimal degrees, using the spatial reference system given in geodeticDatum) of the geographic centre of a Location.
identificationQualifier	A brief phrase or a standard term ("cf.", "aff.") to express the determiner's doubts about the Identification.
scientificNameID	An identifier for the nomenclatural (not taxonomic) details of a scientific name (i.e. AphiaID).
scientificName	The full scientific name.
taxonRank	The taxonomic rank of the most specific name in the scientificName.
taxonRemarks	Comments or notes about the taxon or name.

## Figures and Tables

**Table 1.** Checklist of benthic amphipod species in the Alacranes Reef, Yucatan.

Suborder	Family	Species
Amphilochidea	Ampeliscidae	*Ampelisca brevisimulata* Barnard, 1954
		*Ampelisca cristata* Holmes, 1908
		*Ampelisca schellenbergi* Shoemaker, 1933
		*Ampelisca vadorum* Mills, 1963
	Amphilochidae	*Apolochus delacaya* (McKinney, 1978)
		*Apolochus* sp.
	Bateidae	*Batea carinata* (Shoemaker, 1926)
		*Batea cuspidata* (Shoemaker, 1926)
	Cyproideidae	*Hoplopheonoides obesa* Shoemaker, 1956
	Dexaminidae	*Dexaminella* sp.
		*Notrotopis minikoi* (Walker, 1905)
	Leucothoidae	*Anamixis cavatura* Thomas, 1997
		*Anamixis vanga* Thomas, 1997
		*Leucothoe ashleyae* Thomas & Klebba, 2006
		*Leucothoe barana* Thomas & Klebba, 2007
		*Leucothoe hendrickxi* Winfield & Álvarez, 2009
		*Leucothoe kensleyi* Thomas & Klebba, 2005
		*Leucothoe laurensi* Thomas & Ortiz, 1995
		*Leucothoe saron* Thomas & Klebba, 2007
		*Leucothoe ubouhu* Thomas & Klebba, 2007
		*Leucothoe wuriti* Thomas & Klebba, 2007
		*Leucothoe* sp.
	Liljeborgiidae	*Liljeborgia bousfieldi* McKinney, 1979
		*Liljeborgia* sp.
		*Idunella barnardi* (Wigley, 1966)
		*Idunella* sp.
	Lysianassidae	*Aruga holmesi* Barnard, 1955
		*Concarnes concavus* (Shoemaker, 1933)
		*Ensayara entrichoma* Gable & Lazo-Wasem, 1990
		*Hippomedon* sp.
		*Lepidepecreum madagascarensis* (Ledoyer, 1986)
		*Lysianopsis alba* (Holmes, 1905)
		*Orchomenella thomasi* Lowry & Stoddart, 1997
		*Shoemakerella cubensis* (Stebbing, 1897)
	Oedicerotidae	*Americhelidium americanum* (Bousfield, 1973)
		*Hartmanodes nyei* (Shoemaker, 1933)
		*Perioculodes cerasinus* Thomas & Barnard, 1985
	Phoxocephalidae	*Eobrolgus spinosus* (Holmes, 1905)
		*Harpinia* sp.
		*Metharpinia floridana* (Shoemaker, 1933)
		*Rhepoxynius* sp.
	Platyischnopidae	*Eudevenopus honduranus* Thomas & Barnard, 1983
	Sebidae	*Seba tropica* McKinney, 1980
	Stenothoidae	*Stenothoe gallensis* Walker, 1904
		*Stenothoe valida* Dana, 1853
		*Stenothoe* sp.
Colomastigidea	Colomastigidae	*Colomastix denticornis* LeCroy, 1995
		*Colomastix falcirama* LeCroy, 1995
		*Colomastix heardi* LeCroy, 1995
		*Colomastix irciniae* LeCroy, 1995
		*Colomastix tridentata* LeCroy, 1995
		*Colomastix* sp.
Senticaudata	Ampithoidae	*Ampithoe marcuzzii* Ruffo, 1954
		*Ampithoe ramondi* Audouin, 1826
		*Ampithoe valida* Smith, 1873
		*Cymadusa compta* (Smith, 1873)
		*Cymadusa filosa* Savigni, 1816
		*Pseudamphithoides incurvaria* (Just, 1977)
	Aoridae	*Bemlos dentischium* (Myers, 1977)
		*Bemlos kunkelae* (Myers, 1977)
		*Bemlos longicornis* Myers, 1978
		*Bemlos spinicarpus* (Pearse, 1912)
		*Bemlos unicornis* (Bynum & Fox, 1977)
		*Bemlos* sp.
		*Globosolembos smithi* (Holmes, 1905)
		*Grandidierella bonnieroides* Coutiere, 1904
		*Lembos unifasciatus reductus* Myers, 1979
		*Lembos unifasciatus unifasciatus* Myers, 1977
		*Paramicrodeutopus myersi* (Bynum & Fox, 1977)
		*Plesiolembos ovalipes* (Myers, 1979)
		*Plesiolembos rectangulatus* (Myers, 1977)
	Caprellidae	*Deutella caribensis* Guerra-García, Krapp-Schickel & Müller, 2006
		*Hemiaegina minuta* Mayer, 1890
		*Metaprotella hummelincki* (McCain, 1968)
	Eriopisidae	*Netamelita tabaci* Thomas & Barnard, 1991
		*Psammogammarus* sp.
	Hadziidae	*Protohadzia schoenerae* (Fox, 1973)
	Hornelliidae	Hornellia (Metaceradocus) tequestae Thomas & Barnard, 1986
	Hyalidae	*Parhyale hawaiensis* (Dana, 1853)
		Protohyale (Protohyale) macrodactyla (Stebbing, 1899)
	Ischyroceridae	Ambicholestes (Ambicholestes) crassicornis (Just, 1984)
		*Ericthonius punctatus* (Bate, 1857)
		*Ericthonius* sp.
	Maeridae	*Anamaera hixoni* Thomas & Barnard, 1985
		*Ceradocus rubromaculatus* (Stimpson, 1856)
		Ceradocus (Denticeradocus) sheardi Shoemaker, 1948
		*Ceradocus shoemakeri* Fox, 1973
		*Elasmopus rapax* (Costa, 1853)
		*Elasmopus thomasi* Ortiz & Lalana, 1994
		*Elasmopus* sp.
		*Maera* sp.
		*Maeracoota galani* Krapp-Schickel & Ruffo, 2001
		*Meximaera diffidentia* Barnard, 1969
		*Quadrimaera pacifica* (Schellenberg, 1938)
		*Spathiopus looensis* Thomas & Barnard, 1985
	Megaluropidae	*Gibberosus myersi* (McKinney, 1980)
	Melitidae	*Dulichiella lecroyae* Lowry & Springthorpe, 2007
		*Melita planaterga* Kunkel, 1910
	Neomegamphopidae	*Neomegamphopus hiatus* Barnard & Thomas, 1987
	Nuuanuidae	*Nuuanu muelleri* Ortiz, 1976
	Phliantidae	*Pariphinotus seclusus* (Shoemaker, 1933)
	Photidae	*Audulla chelifera* Chevreux, 1901
		*Gammaropsis sutherlandi* Nelson, 1980
		*Photis* sp.
		*Rocasphotis* sp.
	Podoceridae	*Podocerus fissipes* Serejo, 1995
		*Podocerus kleidus* Thomas & Barnard, 1992
	Pontogeneiidae	*Nasageneia yucatanensis* (McKinney, 1980)
	Talitridae	*Tethorchestia antillensis* Bousfield, 1984
	Unciolidae	*Rudilemboides naglei* Bousfield, 1973

## References

[B3807078] CONANP (2006). Programa de conservación y manejo. Parque Nacional Arrecife Alacranes, Mexico.

[B3993690] González-Muñoz Ricardo, Simões Nuno, Tello-Musi José Luis, Rodríguez Estefanía (2013). Sea anemones (Cnidaria, Anthozoa, Actiniaria) from coral reefs in the southern Gulf of Mexico. ZooKeys.

[B3807171] LeCroy S. E. (2000). An Illustrated Identification Guide to the Nearshore Marine and Estuarine Gammaridean Amphipoda of Florida. Volume 1. Families Gammaridae, Hadziidae, Isaeidae, Melitidae and Oedicerotidae.

[B3807241] Liceaga-Correa M. A., Hernández-Núñez H. (2000). Localización y dimensiones del arrecife Alacranes. Jaina.

[B3807282] Lowry J. K., Stoddart H. E. (1997). Amphipoda
Crustacea IV. Families Aristiidae, Cyphocarididae, Endevouridae, Lysianassidae, Scopelocheiridae, Uristidae. Memoirs of the Hourglass Cruises.

[B3993757] Lowry J. K., Myers A. A. (2017). A phylogeny and classification of the Amphipoda with the establishment of the new order Ingolfiellida (Crustacea: Peracarida). Zootaxa.

[B3807314] Myers A. A. (1981). Amphipod Crustacea I, Family Aoridae. Memoirs of the Hourglass Cruises.

[B3807364] Ortiz M. T., Winfield I. A., Scheinvar G. E., Cházaro O. S. (2014). Clave Ilustrada de anfípodos del Golfo de México y el Mar Caribe (Gammaridea y Caprellidea).

[B3807373] Paz-Ríos C. E., Simões N., Ardisson P. L. (2013). Records and observations of amphipods (Amphipoda: Gammaridea and Corophiidea) from fouling assemblages in the Alacranes Reef, southern Gulf of Mexico. Marine Biodiversity Record.

[B3807403] Paz-Ríos C. E., Simões N., Ardisson P. L. (2013). Intertidal and shallow water amphipods (Amphipoda: Gammaridea and Corophiidea) from Isla Pérez, Alacranes Reef, southern Gulf of Mexico. Nauplius.

[B3807413] Paz-Ríos C. E., Guerra-García J. M., Ardisson P. L. (2014). Caprellids (Crustacea: Amphipoda) from the Gulf of Mexico, with observations on *Deutella
mayeri*, redescription of *Metaprotella
hummelincki*, a taxonomic key and zoogeographical comments. Journal of Natural History.

[B3993710] Ramírez-Hernández Adriana, Hernández-Alcántara Pablo, Solís-Weiss Vivianne (2015). *Nereis
alacranensis*, a new species of polychaete (Annelida, Nereididae) from Alacranes Reef, southern Gulf of Mexico, with a key to *Nereis* from the Grand Caribbean. Zootaxa.

[B3993745] Reyes-Gómez Adriana, Ortigosa Deneb, Simões Nuno (2017). Chitons (Mollusca, Polyplacophora) from Alacranes Reef, Yucatan, Mexico. ZooKeys.

[B3993700] Santana-Moreno Luis Daniel, Grave Sammy De, Simões Nuno (2013). New records of caridean shrimps (Decapoda: Caridea) from shallow water along the northern Yucatan Peninsula coasts of México. Nauplius.

[B3913069] Tunnell J. W., Chávez E. A., Withers K. (2007). Coral Reefs of the Southern Gulf of Mexico.

[B3913078] Tunnell J. W., Barrera N., Beaver C. R., Davidson J., Gourley J. E., Moretzsohn F., Nañez-James S., Pearce J. J., Vega M. E. Checklist of the biota associated with Southern Gulf of Mexico coral reefs and coral reef islands. http://www.gulfbase.org/checklist/.

[B3993730] Ugalde Diana, Gómez Patricia, Simões Nuno (2015). Marine sponges (Porifera: Demospongiae) from the Gulf of México, new records and redescription of *Erylus
trisphaerus* (de Laubenfels, 1953). Zootaxa.

[B3807563] Wilkinson T., Wiken E., Bezaury-Creel J., Hourigan T., Agardy T., Herrmann H., Janishevski L., Madden C., Morgan L., Padilla M. (2009). Marine Ecoregions of North America.

[B3807578] Winfield I., Escobar-Briones E., Álvarez F. (2007). Clave para la identificación de los anfípodos bentónicos del Golfo de México y el sector norte del Mar Caribe.

